# Is sunlight an aetiological agent in the genesis of retinoblastoma?

**DOI:** 10.1038/sj.bjc.6690204

**Published:** 1999-03

**Authors:** M L Hooper

**Affiliations:** Sir Alastair Currie CRC Laboratories, Department of Pathology, Molecular Medicine Centre, University of Edinburgh, Crewe Rd, Edinburgh, EH4 2XU, UK

**Keywords:** retinoblastoma, epidemiology, sunlight, ultraviolet radiation

## Abstract

The incidence of unilateral, but not bilateral, retinoblastoma in human populations at different geographical locations increases significantly with ambient erythemal dose of ultraviolet B radiation from sunlight. This supports the hypothesis that sunlight plays a role in retinoblastoma formation. © 1999 Cancer Research Campaign


*British Journal of Cancer* (1999) **79**(7/8), 1273–1276
© 1999 Cancer Research Campaign
Article no. bjoc.1998.0204

### Is sunlight an aetiological agent in the genesis of retinoblastoma?

**ML Hooper**

Sir Alastair Currie CRC Laboratories, Department of Pathology, Molecular Medicine Centre, University of Edinburgh, Western General Hospital, Crewe Rd, Edinburgh EH4 2XU, UK

**Summary** The incidence of unilateral, but not bilateral, retinoblastoma in human populations at different geographical locations increases significantly with ambient erythemal dose of ultraviolet B radiation from sunlight. This supports the hypothesis that sunlight plays a role in retinoblastoma formation.

**Keywords:** retinoblastoma; epidemiology; sunlight; ultraviolet radiation

Retinoblastomas are childhood retinal tumours of which about 40% of all cases are familial (reviewed by Knudson, 1993). Familial retinoblastoma cases are usually bilateral, unlike sporadic cases in which only one eye is normally affected. Knudson (1971) proposed that retinoblastomas arise as a result of two mutational events, of which one is present in the germline in familial cases. Recent molecular analysis has confirmed that this is indeed the case, and demonstrated that both events involve modification or loss of an allele of the same gene, *RB-1*, which is commonly regarded as the paradigm for oncosuppressor genes (Mittnacht, 1998). More than 90% of individuals constitutively heterozygous for an *RB-1* mutation develop retinoblastoma as a result of a somatic event occurring in one or more cells of the retina, or of its precursor tissues, that eliminates the function of the wild-type allele, usually by allele loss (Knudson, 1993).

Three different null alleles of the corresponding murine gene, designated *Rb-1*, have been generated independently by gene targeting (reviewed by Hooper, 1994). In contrast to the situation in humans, no retinoblastomas have to date been detected in mice heterozygous for any of the null alleles of *Rb-1*. There are a number of possible explanations for this (Hooper, 1994), of which one is that laboratory mice differ from humans in the level of exposure of their retinas to sunlight. Here, I have analysed retinoblastoma incidence at geographical locations differing in sunlight exposure to test the hypothesis that sunlight plays a role in its causation.

### MATERIALS AND METHODS

Cumulative incidence of retinoblastoma for children of ages 0–14 at different geographical locations during various time intervals between 1969 and 1987 were taken from Parkin et al (1988), Parkin and Stiller (1995), and from data used in the compilation of Parkin et al (1992) which were communicated by Dr Max Parkin. For each population under study, mean values of latitude and longitude were estimated, and these were used to determine the annual ambient erythemal dose of UVB by the method of Diffey and Elwood (1993) using tabulated dose values for clear skies at each latitude and plots of average percentage cloud cover as a function of latitude and longitude, which they provide, at intervals throughout the year and using linear interpolation between tabulated and plotted values. For the purposes of this analysis, cases of eye tumour with unspecified histology were distributed between retinoblastoma and other tumour types in the same ratio as the known cases for the same centre, and this accounts for entries showing fractional numbers of cases. The data were analysed by linear regression, weighted to take account of population size, using the GLIM statistical package (Numerical Algorithms Group, Oxford, UK).

*Received 2 July 1998*
*Revised 31 July 1998*
*Accepted 5 August 1998*

*Correspondence to:* ML Hooper

### RESULTS

In a preliminary analysis, I have reported that published values of the incidence of retinoblastoma taken from Parkin et al (1988) fall significantly with increasing geographical latitude (Hooper, 1994), consistent with an aetiological role for sunlight. However, because of the earth's axial tilt, sunlight exposure is greatest at the tropics rather than at the equator, and it is also influenced by cloud cover (Diffey and Elwood, 1993). I have, therefore, refined the analysis (Table 1 and Figure 1) using annual ambient erythemal doses of UVB calculated from published tables. This analysis shows that incidence does indeed increase significantly with dose, the difference between the slope of the regression line and zero being significant at the 1% level.

Most centres do not report on the laterality of tumours. However, analysis of data from those centres that do report laterality (Table 2 and Figure 2) shows no significant increase in the incidence of bilateral tumours with annual ambient UV dose, but a highly significant one in the case of unilateral tumours.

**Table 1** Incidence of retinoblastoma at different locations

| Location | Latitude | Annual ambient erythemal dose (MED year$^{-1}$) | Number of cases studied (0–14 years) | Cumulative incidence (cases per million) |
|---|---|---|---|---|
| Denmark | 56°N | 1450 | 26.8 | 62 |
| England and Wales | 52°N | 1620 | 363.9 | 42 |
| Estonia | 59°N | 1270 | 4 | 34 |
| Finland | 61°N | 1140 | 58 | 59 |
| France | 47°N | 2080 | 20 | 44 |
| German Democratic Republic | 52°N | 1670 | 48 | 48 |
| German Federal Republic | 51°N | 1740 | 78 | 44 |
| Hungary | 47°N | 2080 | 34 | 22 |
| Italy | 44°N | 2560 | 36 | 61 |
| Netherlands | 52°N | 1650 | 26 | 63 |
| Norway | 61°N | 1100 | 26 | 43 |
| Poland, Warsaw | 52°N | 1690 | 12 | 53 |
| Scotland | 56°N | 1350 | 59 | 55 |
| Slovakia | 49°N | 1940 | 81.8 | 62 |
| Slovenia | 46°N | 2140 | 17 | 41 |
| Spain | 40°N | 2890 | 12 | 54 |
| Sweden | 59°N | 1270 | 87 | 64 |
| Switzerland | 47°N | 2070 | 8 | 32 |
| China, Shanghai | 31°N | 3450 | 13 | 39 |
| Hong Kong | 22°N | 4160 | 22 | 46 |
| Israel (Jews) | 32°N | 4320 | 35 | 35 |
| Israel (non-Jews) | 32°N | 4320 | 9 | 45 |
| India, Bangalore | 13°N | 5450 | 10 | 42 |
| India, Bombay | 19°N | 5160 | 103 | 70 |
| Japan | 35°N | 2990 | 177 | 59 |
| Kuwait (Kuwaitis) | 29°N | 4910 | 5 | 31 |
| Kuwait (non-Kuwaitis) | 29°N | 4910 | 8 | 53 |
| Philippines | 15°N | 4800 | 136.9 | 93 |
| Singapore | 1°N | 4700 | 40 | 52 |
| Taiwan, Taipei | 25°N | 3940 | 22 | 27 |
| Thailand | 14°N | 5010 | 32.9 | 44 |
| Nigeria, Ibadan | 7°N | 5010 | 14 | 102 |
| Uganda | 0° | 5340 | 29 | 101 |
| Zimbabwe | 20°S | 5340 | 16 | 85 |
| Canada, western provinces | 52°N | 1720 | 90.9 | 58 |
| Canada, Atlantic provinces | 46°N | 2100 | 72.5 | 50 |
| Cuba | 22°N | 4880 | 120 | 48 |
| Jamaica | 18°N | 5400 | 13 | 62 |
| Puerto Rico | 18°N | 5140 | 68 | 54 |
| Atlanta (whites) | 34°N | 3340 | 10 | 61 |
| Connecticut (whites) | 42°N | 2290 | 15 | 41 |
| Detroit (whites) | 42°N | 2430 | 20 | 50 |
| Greater Delaware Valley (whites) | 40°N | 2580 | 63 | 65 |
| Greater Delaware Valley (non-whites) | 40°N | 2580 | 19 | 83 |
| Iowa (whites) | 42°N | 2500 | 31 | 70 |
| Los Angeles (blacks) | 34°N | 3600 | 13 | 65 |
| Los Angeles (whites) | 34°N | 3600 | 24 | 48 |
| New Mexico (whites) | 35°N | 3660 | 8 | 37 |
| New York (blacks) | 42°N | 2290 | 10 | 35 |
| New York (whites) | 42°N | 2290 | 46 | 35 |
| San Francisco (whites) | 38°N | 3110 | 20 | 67 |
| Seattle (whites) | 48°N | 1960 | 26 | 69 |
| Utah (whites) | 40°N | 2920 | 23 | 60 |
| Brazil, Fortaleza | 4°S | 5750 | 12 | 111 |
| Brazil, Recife | 8°S | 5540 | 27 | 68 |
| Brazil, Sao Paulo | 24°S | 4880 | 108 | 78 |
| Colombia, Cali | 4°N | 4880 | 12 | 88 |
| Costa Rica | 10°N | 5160 | 30 | 98 |
| Australia, New South Wales | 34°S | 3810 | 76 | 55 |
| Australia, Queensland | 25°S | 4850 | 25 | 73 |
| South Australia | 34°S | 3970 | 5 | 49 |
| Australia, Victoria | 37°S | 3180 | 14 | 46 |
| Western Australia | 32°S | 3860 | 10 | 87 |
| Fiji (Fijians) | 18°S | 4790 | 6 | 50 |
| Fiji (Indians) | 18°S | 4790 | 4 | 28 |
| New Zealand | 41°S | 2740 | 72 | 85 |

Retinoblastoma incidence data are taken from Parkin et al (1988) and from data used in the compilation of Parkin et al (1992).

### DISCUSSION

The effects on retinoblastoma incidence documented here, even when unilateral cases are considered separately, are less severe than those reported for squamous cell carcinoma of the eye, the incidence of which varies over more than two orders of magnitude between centres with high and low UVB exposure (Newton et al, 1996) and which may explain why they have not previously been detected.

A number of possible reasons must be considered for the effect seen in Figure 1. First, it could be a consequence of ascertainment bias, due either to variations in under- or over-reporting of retinoblastoma or in estimating the size of the population from which the patients are drawn. It might, for instance, be argued that data from equatorial regions are least reliable in this context because they contain a higher proportion of information from developing countries. Second, it could be due to genetic differences between the populations under study in the form of differences in frequency of germline *RB-1* mutations, or differences in frequency of alleles at other loci that influence the frequency of somatic events leading to loss of heterozygosity at the *RB-1* locus resulting, for instance, from variations in the racial composition of populations. Third, it could be a direct effect of exposure to UVB, or to other wavelengths present in sunlight, or to another environmental variable correlated with sunlight exposure; the most obvious hypothesis is that it is due to an effect of sunlight on somatic events leading to *RB-1* inactivation. Based on this hypothesis, because individuals inheriting a germline *RB-1* mutation almost invariably develop bilateral retinoblastomas, one would predict that the effect would be due largely or entirely to unilateral tumours, which in general occur sporadically and require two somatic events. The data of Figure 2 are consistent with this prediction.

The analysis shown in Figure 2 argues against the hypothesis that the effect in Figure 1 is due to ascertainment bias, which would be expected to affect data on unilateral and bilateral tumours in a similar fashion; it also argues that it is not due to differences in *RB-1* germline mutation frequency, which would

 *© Cancer Research Campaign 1999*

**Table 2** Incidence of unilateral and bilateral retinoblastoma at different locations

| Location | Annual ambient erythemal dose (MED year$^{-1}$) | Number of retinoblastoma cases studied (0–14 years) | Cumulative incidence of unilateral retinoblastoma (cases per million) | Cumulative incidence of bilateral retinoblastoma (cases per million) |
|---|---|---|---|---|
| Great Britain | 1620 | 699 | 27.4 | 16.6 |
| India, Bombay | 5160 | 63 | 59 | 10 |
| Japan, Osaka | 3020 | 56 | 50 | 18 |
| Nigeria, Ibadan | 5010 | 97 | 75 | 25 |
| South Africa (blacks) | 4610 | 78 | 80 | 18 |
| USA, Greater Delaware Valley (blacks) | 2580 | 19 | 62 | 17 |
| USA, Greater Delaware Valley (whites) | 2580 | 63 | 37 | 21 |
| USA (Navajo Indians) | 3660 | 15 | 125 | 28 |
| USA, SEER[a] registry (blacks) | 2790 | 28 | 43 | 17 |
| USA, SEER registry (whites) | 2790 | 167 | 41 | 13 |
| Australia, Queensland | 4850 | 25 | 59 | 22 |

[a]Data from nine population-based registries in different parts of the USA which have no overlap with any other data listed in this table. Incidence data are taken from Parkin and Stiller (1995) and references cited therein. No other independent datasets in which incidences of unilateral and bilateral retinoblastoma were recorded separately were identified in a literature search.

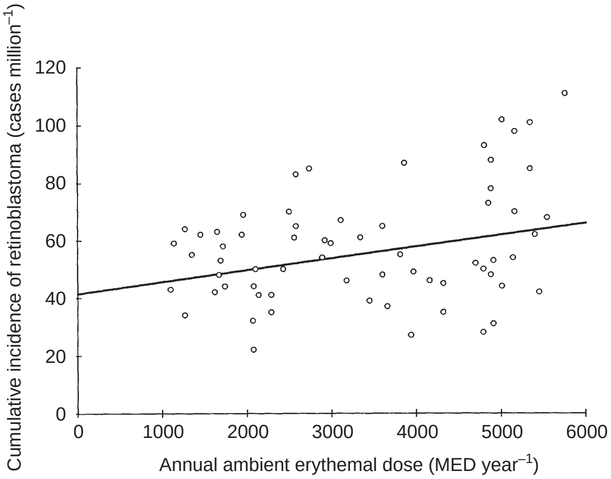

**Figure 1** Cumulative incidence of retinoblastoma for children of ages 0–14 for the locations listed in Table 1, plotted as a function of annual ambient erythemal dose of UVB. The bold continuous line is a linear regression, whose slope, 0.004100 ± s.e. 0.001334, is significantly different from zero ($t_{64}$ = 3.073, 0.001 < $P$ < 0.01)

affect bilateral tumour incidence preferentially. The hypotheses that it is due to differences in allele frequencies at other loci, or to an environmental variable correlated with sunlight exposure, cannot be formally excluded, but a direct effect of sunlight provides the most ready explanation of the data. This would be consistent with the mutation spectrum of sporadic retinoblastoma: first-hit lesions include some C to T transitions, consistent with action of UV radiation, although instances of the CC to TT change that is more diagnostic of UV mutagenesis have not been reported, whereas second-hit lesions are uninformative usually involving gross chromosomal change (reviewed by Murphree and Munier, 1994). To test this hypothesis further, it would be valuable for more centres to report the laterality of tumours, and also to report eye pigmentation so that its effect on incidence can be assessed. It would also be of interest to determine the critical period for UV exposure. Most retinoblastomas develop in the first 5 years of life (Knudson, 1971) and the period of susceptibility to UV may be restricted to a relatively small fraction of this 5-year interval, perhaps immediately after birth when the retina is least mature. If this period is less than a year, it may be possible to detect a variation in retinoblastoma incidence at a given location as a function of the month of birth. If not, a study of patients who had migrated between locations with different UV exposure would be necessary, posing daunting problems in defining a sufficiently large dataset and in identifying and quantifying the corresponding ‘at risk’ populations.

We have tested the hypothesis that differences in exposure to ultraviolet radiation between mice and humans are alone sufficient to account for the lack of retinoblastomas in *Rb-1*$^{+/-}$ mice by controlled exposure of the mice to fluorescent light with a daylight spectrum, but have not observed any retinoblastomas in the exposed mice (JF Armstrong, MH Kaufman and MLH, unpublished observations). This leaves open the possibility that it is important in combination with other differences. The latter could include a possible need in the mouse for an additional genetic event or events involving genes such as *p53*, *p107* or *p130* (Hooper, 1994). We have carried out controlled exposure of *Rb-1*$^{+/-}$ p53$^{-/-}$ mice to light without obtaining any retinoblastomas. However, it has recently been reported (Robanus Maandag et al, 1998) that retinoblastomas developed in 6 out of 14 chimaeric eyes in mice containing cells doubly homozygous for mutant alleles of

© Cancer Research Campaign 1999 

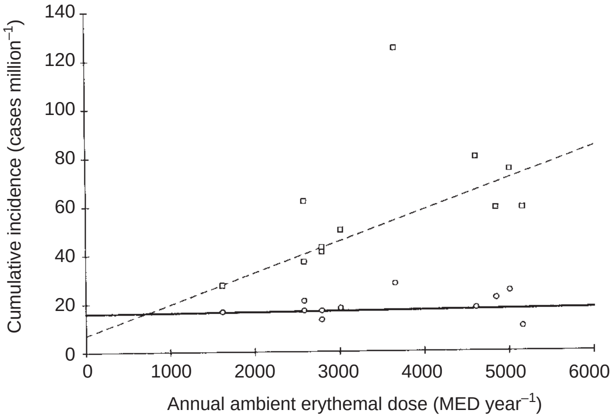

**Figure 2** Cumulative incidence of unilateral (squares) and bilateral (circles) retinoblastoma for children of ages 0–14 at the locations listed in Table 2, plotted as a function of annual ambient erythemal dose of UVB. Regression lines have been fitted separately to unilateral data: broken line, slope 0.01295 ± s.e. 0.001958, significantly different from zero ($t_9$ = 6.614, $P$ < 0.001); and to bilateral data, bold continuous line, slope 0.0003361 + s.e 0.0008433, not significantly different from zero ($t_9$ = 0.3986, $P$ > 0.5)

*Rb-1* and *p107*. This demonstrates that in addition to *Rb-1* inactivation, retinoblastoma formation in the mouse requires mutation of *p107* and probably a further event involving an unidentified gene that occurred somatically in the chimaeras. There is not, at present, a ready explanation for a difference in requirement for these additional events between mouse and human. Nonetheless, whatever the complete explanation for the difference in retinoblastoma incidence between *Rb-1*$^{+/-}$ mice and *RB-1*$^{+/-}$ humans, it has stimulated an analysis that has revealed a previously unsuspected association between UVB exposure and the incidence of unilateral, but not bilateral, retinoblastoma in human populations.

### ACKNOWLEDGEMENTS

I am grateful to Max Parkin for helpful discussion and for communication of retinoblastoma incidence data, and to Jane Armstrong, Matt Kaufman and Anton Berns for helpful discussion. Work on mutant mice in my laboratory was supported by the Medical Research Council and the Cancer Research Campaign.

### REFERENCES

Diffey BL and Elwood JM (1993) Tables of ambient solar ultraviolet radiation for use in epidemiological studies of malignant melanoma and other diseases. In *Melanoma Epidemiology*. Gallacher R and Elwood JM (eds), pp. 81–105. Kluwer: New York

Hooper ML (1994) The role of the *p53* and *Rb-1* genes in cancer, development and apoptosis. *J Cell Sci* **18** (*Suppl*): 13–17

Knudson AG (1971) Mutation and cancer: statistical study of retinoblastoma. *Proc Natl Acad Sci USA* **68**: 820–823

Knudson AG (1993) Antioncogenes and human cancer. *Proc Natl Acad Sci USA* **90**: 10914–10921

Mittnacht S (1998) Control of pRb phosphorylation. *Curr Opin Genet Dev* **8**: 21–27

Murphree AL and Munier FL (1994) Retinoblastoma. In *Retina*, 2nd edn. Ryan SJ (editor-in-chief), pp. 571–586. Mosby: St. Louis

Newton R, Ferlay J, Reeves G, Beral V and Parkin DM (1996) Effect of ambient solar ultraviolet radiation on incidence of squamous-cell carcinoma of the eye. *Lancet* **347**: 1450–1451

Parkin DM and Stiller CA (1995) Childhood cancer in developing countries: environmental factors. *Int J Pediat Hematol Oncol* **2**: 411–417

Parkin DM, Stiller CA, Bieber A, Draper GJ, Terracini B and Young JL (1988) *International Incidence of Childhood Cancer*. IARC Scientific Publication no. 87. IARC: Lyon

Parkin DM, Muir CS, Whelan SL, Gao Y-T, Ferlay J and Powell J (1992) *Cancer Incidence in Five Continents*, Vol. VI. IARC Scientific Publication no. 120. IARC: Lyon

Robanus Maandag E, Dekker M, van der Valk M, Carrozza M-L, Jeanny J-C, Dannenberg J-H, Berns A and te Riele H (1998) p107 is a suppressor of retinoblastoma development in pRb-deficient mice. *Genes Dev* **12**: 1599–1609

 *© Cancer Research Campaign 1999*
